# Bilateral symmetry and anatomical root variations of mandibular second molars in North Indian populations using cone beam computed tomography

**DOI:** 10.4317/jced.60063

**Published:** 2023-02-01

**Authors:** Rakesh-Kumar Yadav, Jyoti Tandon, Neha Jasrasaria

**Affiliations:** 1 Professor. Conservative Dentistry and Endodontics KGMU, Lucknow; 2 Senior Resident. Conservative Dentistry and Endodontics KGMU, Lucknow

## Abstract

**Background:**

To evaluate bilateral symmetry and anatomical variations of root morphology of permanent mandibular second molar using systematic evaluation of CBCT scans.

**Material and Methods:**

This cross-sectional study involved the mandible which were imaged using serial axial cone-beam computed tomography (CBCT) from 680 North Indian patients who visited dental hospital for various reasons unrelated to the study. CBCT Records with presence of bilateral fully erupted permanent mandibular second molars with fully formed apexes were selected.

**Results:**

Bilaterally present two root and three canal configurations were most consistently seen (75.88 % and 59.11 %, respectively). The occurrence of two roots with two canals and four canals was 15.14% and 1.61% respectively. Mandibular second molar present with one extra root, radix entomolaris with three canals and four canals was 0.44% and 3.53% while radix paramolaris present with three canals and four canals 1.32% and 1.03% respectively. The occurrence of C-shaped root bilaterally with C shaped canal was 15.88% whereas the presence of one fused root bilaterally was 0.44%. The presence of four roots bilaterally with four canals was identified in only one CBCT scanned image (0.14%). The frequency distribution of root morphology on bilateral symmetrical analysis revealed 98.58 % bilateral symmetry.

**Conclusions:**

In 402 CBCT scans, bilateral presence of two roots with three canals was the most typical root structure seen in mandibular second molars (59.11%). A rare variation found was the presence of four roots occurring bilaterally, seen in only 1 CBCT scan. Bilateral symmetrical analysis of root morphology revealed 98.58 % bilateral symmetry.

** Key words:**Anatomic root variations, mandibular second molar, Cone Beam Computed Tomography scans, bilateral symmetry.

## Introduction

Mandibular second molar is a tooth with major concern as they are mostly affected by distal caries due to impacted third molars and its anatomy is known to be highly variable. The highest incidence of caries in mandibular second molar has been reported by Van der Linden *et al*. as 32% (1) whereas Nur Altiparmak *et al*., reported 39% distal caries lesions (2).

The most important factor for success of any treatment depends upon proper diagnosis. A mandibular second molar consists of a large number of variations which may be challenging to the clinician. The proper diagnosis of multirooted teeth requires evaluation of morphology and anatomy of roots with the knowledge of their possible variations reported in the previous literature (3). One of the most common factors of endodontic retreatment is missed canal. There is a considerable probability of missing anatomy during root canal treatment because of how complex the root canal system is and how different root morphologies can be. All of the permanent teeth in humans may have additional roots or canals, although premolars and molars are more likely to have unusual canal configurations (4).

Various techniques are available for diagnosis and study of tooth anatomy such as conventional periapical radiography and computed tomography. Conventional two-dimensional radiographs have shortcomings such as overlapping of bony and dental structures, distortion of image and two-dimensional representation of 3D object. Recently, Cone beam computerized tomography (CBCT) has been introduced in dentistry for imaging hard tissues of the maxillofacial region, available as most recent imaging technique (5). As a non-invasive technology, CBCT offers benefits since it can provide three-dimensional pictures of better spatial resolution. CBCT has advantages over conventional radiography as it has a combination of axial, coronal and sagittal sections, which reduces distortion of images and overlapping of anatomical structures providing the clinicians more accurate anatomy of tooth structure and enhanced visualization of area of interest. CBCT when compared with conventional computed tomography (CT) has lesser radiation dose, lower scanning time and higher resolution (6). In order to evaluate the morphology of the second molar root anatomy in the mandible, CBCT was performed in the current study.

Identification of the root morphologies and variations of various races is necessary because root morphology and its variants may have a definite racial influence (7). A vast literature search revealed that there is only one study on root morphology and its variations of mandibular second molars in an Indian population (8). However, there are no reports on root morphology variation in mandibular second molars occurring with four roots in an Indian population. The aim of the study was to evaluate anatomical root variations and to determine bilateral symmetry of root morphological aberrations in mandibular second molar. The study was also used to determine the relative incidence of bilaterally four rooted mandibular second molars, a rare anatomic variation in North Indian population using systematic evaluation of cone beam computed tomography (CBCT) scans.

## Material and Methods

-Subjects:

Cone-beam computed tomography (CBCT) scans of the mandibles from 680 North Indian patients, 364 men and 316 females, aged between 17 years and 60 years, were collected and screened, who visited the Department of Oral Medicine and Radiology; Faculty of Dental Science, KGMU, Lucknow between June 2017 - July 2019. Unrelated to the current investigation, these individuals had CBCT imaging as part of their therapy and for a variety of diagnostic objectives. A total of 830 individuals had their CBCT scans examined, and 680 of them were found to match the requirements for CBCT Records by having bilaterally present, completely erupted mandibular second molars with fully developed apexes.

-CBCT evaluation:

Serial axial, coronal, and sagittal plane analyses of CBCT scan pictures were displayed on an LCD monitor. To count the roots and their associated morphologic deviations in permanent mandibular second molar teeth, the toolbar was continuously moved from the bottom to the top of the pulp chamber for detailed analysis of the CBCT pictures. The following CBCT findings were recorded:

i.) Frequency of roots, 

ii.) Frequency of anatomic variations of a root according to the gender, 

iii.) Unilateral or bilateral occurrence of anatomic variations, 

iv.) Incidence of occurrence of rare anatomic variation, presence of four roots in mandibular second molar.

The apical third piece (from the apical third of the canal length to the apical foramen) and middle third piece (from the middle third of the canal to the coronal third) of the scanned cross-sectional canal photographs were also analyzed. Before the experiment, intra-examiner calibration was done to ensure the accuracy of the results. The two residents debated their differing interpretations of the pictures until they came to an agreement with the assistance of a skilled oral radiologist. The photographs were cropped and made larger, which inevitably resulted in pixilation.

Results

-Frequency of Number of Roots

**Figure 1:**
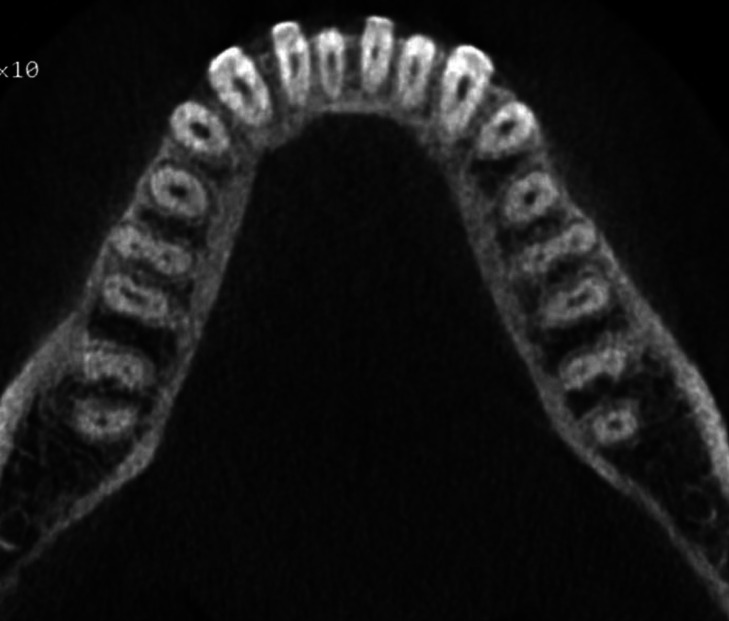
Cross-sectional cone-beam computed tomography image of a mandibular second molar with presence of two roots and three canals occurring bilaterally.

**Figure 2:**
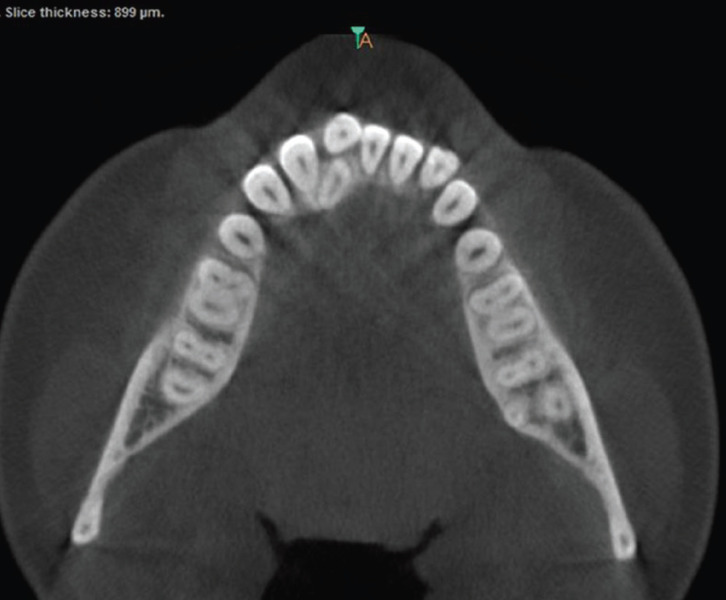
Cross-sectional cone-beam computed tomography image of a mandibular second molar with presence of c-shaped root occurring bilaterally.

**Figure 3:**
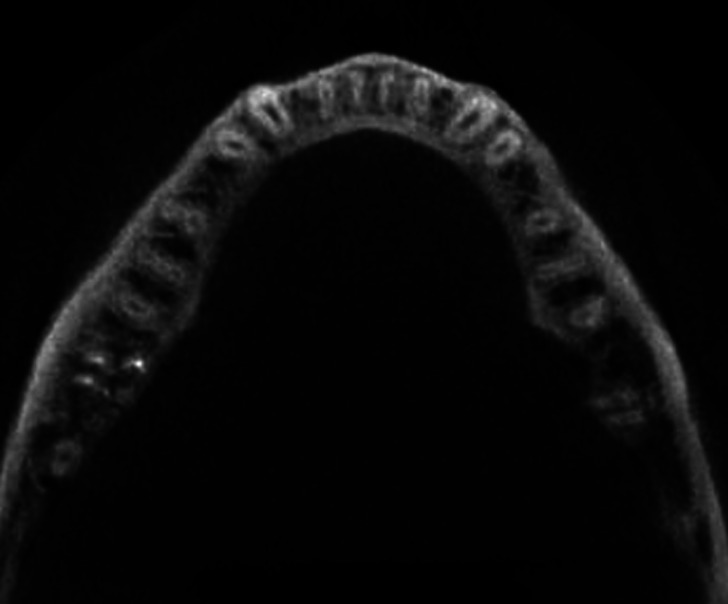
CBCT axial image of 4-rooted second mandibular molars present bilaterally.

**Figure 4:**
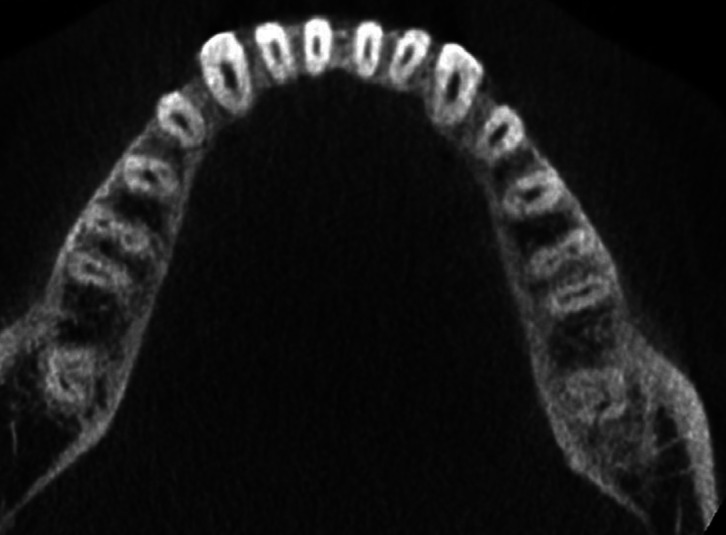
Cross-sectional cone-beam computed tomography image of a mandibular second molar with presence of two roots with three canals (right) occurring unilaterally and three roots with four canals (left).

In 680 CBCT scanned pictures, the number of roots in mandibular second molars were counted. In 516 scanned CBCT pictures, the bilateral presence of two-root was the most prevalent conFiguration (75.88 %). Presence of extra root, three roots in mandibular second molar was present bilaterally in 43 CBCT scans (6.32%) (Fig. 1). The occurrence of extra root distolingually, Radix entomolaris identified in 27 CBCT scans (3.97%). Radix paramolaris presence of extra root mesiobuccally identified in 16 CBCT scans (2.35%) (Fig. 2). Four roots in mandibular second molar was identified in one CBCT scan (0.14%) (Fig. 3). C-shaped fused root was identified in 108 CBCT images (15.88%) (Fig. 4). Mandibular second molar with single root was present in 3 CBCT images (0.44%).

-Frequency of Roots and Their Variations According to Gender.

The number of mandibular second molar roots was assessed in 680 CBCT scans of 364 males and 316 females between the ages of 17 and 65 years. Females were found to have two roots more frequently (39.56%), but males had additional roots and root variants more frequently. Table 1 displays the mandibular second molar’s root morphology’s frequency distribution.

**Table 1:**
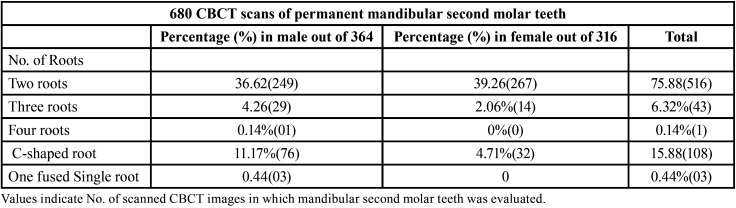
The percentage of frequency of roots evaluated in 680 CBCT scans of permanent mandibular second molar teeth and their distribution in males and females.

-Frequency of Number of Root Canals

In 402 CBCT scan pictures, there are two rooted mandibular second molars, one mesial root with two canals and one distal root with one canal (59.11 percent). In mandibular second molars with two and three roots, different numbers of root canals were discovered. Root canal variations for the fused C-shaped root are presented in Table 2.

**Table 2:**
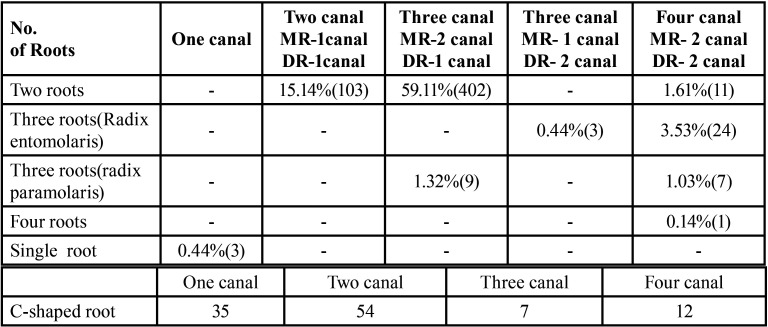
The percentage of frequency of number of root canals in the evaluated 680 CBCT scans of permanent mandibular second molar teeth.

-Bilateral and Unilateral Occurrence of Root Morphology

The frequency distribution of root morphology on bilateral symmetrical analysis revealed 98.58 % bilateral symmetry on right and left side shown in Table 3. Unilateral occurrence of number of roots and their variations shown in Table 4. The unilateral occurrence of two roots with three canals and three roots with three canals (0.58%). The incidence of two roots with three canals and a C-shaped root (0.73%) shown in Table 4.

**Table 3:**
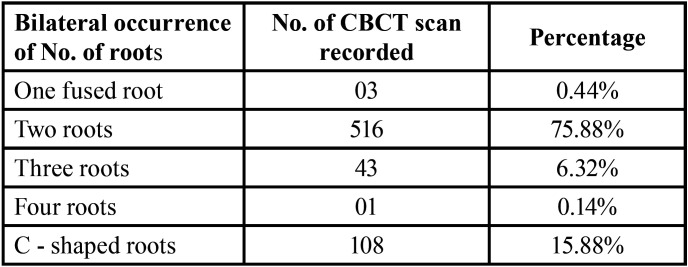
The percentage of bilateral occurrence of root morphology on bilateral symmetrical analysis of mandibular second molar of 680 CBCT.

**Table 4:**
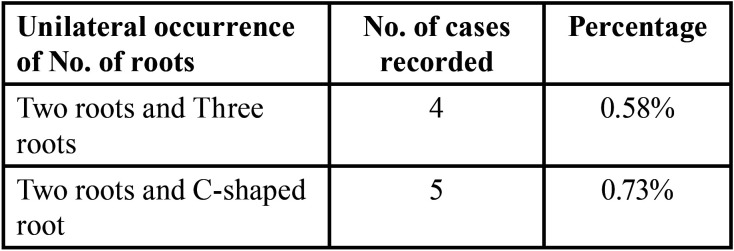
The percentage of unilateral occurrence of root morphology on bilateral symmetrical analysis of mandibular second molar of 680 CBCT.

## Discussion

The mandibles of North Indian patients who visited the dental clinic for a variety of reasons unrelated to the research were scanned using CBCT scans for this investigation. A total of 680 CBCT scans were screened to evaluate anatomical root variations, and bilateral symmetry of root morphology of permanent mandibular second molar in North Indian population. CBCT is a recent three-dimensional radiographic technique used in dentistry for diagnosis and treatment planning. CBCT provides more accurate, high-resolution images of tooth structure and prevents overlapping of anatomical structures 5 thus providing definite tooth anatomy details when compared with conventional radiography. Therefore, in this study CBCT imaging technique was used for anatomical evaluation of mandibular second molar tooth.

Mandibular second molar are tooth presenting high variations in their root morphology. Identifying the usual anatomical morphology and its variances in a tooth may make it easier to spot these deviations while receiving endodontic treatment, increasing the likelihood that the procedure will be successful (7).

The present study emphasized on the bilateral occurrence of root morphology aberrations in mandibular second molar. According to reports, in the Caucasian population mandibular second molars commonly have three root canals and two roots that are mesiodistally positioned8. However, several studies revealed a significant incidence of C-shaped root canals (10-44.5%) in Asian populations, and it has been demonstrated that this prevalence varies greatly by race (9-11).

Neelakantan *et al*. reported mandibular molar with two separate roots (83.4%) which is the highest percentage reported in the literature in Indian population (12).

 In the present study, 680 CBCT scan images screened showed that the most common root morphology of 516 CBCT images (75.88%) of the mandibular second molars had two roots, one mesial root, and one distal root. In the present study, mandibular second molar showing one mesial root with 2 canals and one distal root with one canal (59.11%) was present bilaterally in 402 CBCT scan images. Two rooted mandibular second molar present with one canal in mesial root and one canal in distal root were present in 103 CBCT scans (15.14%) while 2 canals in mesial root and 2 canals in distal root were observed in 11 CBCT scans (1.61%).

In mandibular second molar, most common root morphology reported in literature is presence of two roots with three canals. Pawar *et al*., used CBCT in their study and reported 2 separate roots present mesiodistally (79.35%) in mandibular second molar (13). The data on the root canal morphology of mandibular second molars in the Indian population that have been documented in the literature are not supported by the current findings (12,13). This is more than the prevalence in the Thai (54%) and Burmese (58.2%) populations (14,15).

The mandibular molars have an extra root, which is one of the anatomical distinctions. It is known as the radix entomolaris in the distolingual region or the radix paramolaris in the mesiobuccal location, as initially described in literature by Carabelli (16-18). The occurrence of an extra roots in mandibular molars is associated with certain ethnic groups (19-21).

In the present study, the occurrence of bilateral three rooted mandibular second molar was observed in 43 CBCT scans (6.32%) in an Indian population. Radix entomolaris, presence of three roots with four canals (two canals in mesial root and two canals in distal root) was seen in 24 CBCT scans (3.53%) and radix entomolaris with three canals (one canal in mesial root and two canal in distal root) was seen in 3 CBCT scans (0.44%). Radix paramolaris presence of three canals (two canals in mesial root and one canal in distal root) identified in 9 CBCT scans (1.32%) while paramolaris with four canals (two canals in mesial root and two canals in distal root) were identified in 7 CBCT scans (1.03%). Symmetrical analysis of all 43 CBCT scans revealed symmetry of roots on left and right side.

Presence of extra root in mandibular second molar is not a unique variation when compared with other populations. 7.53% of second molars in the mandible have three roots, according to Pawar *et al*. (13). There have been conflicting findings from research on the mandibular second molars of the Chinese and Israeli populations. One study claimed that none of the teeth had three roots (14) while more recent investigations found that the incidence in the Chinese population was 1.27% (22). In the other study on mandibular second molar by Shemesh *et al* reported the presence of radix entomolaris was 0.41% and the occurrence of radix paramolaris was 1.37% in the Israeli population (23). According to Kim *et al*., 0.72% of mandibular second molars had three roots, which is less than the prevalence of three-rooted mandibular second molars in Thai communities (1.2%) 15 and more than those of Burmese cultures (0%) (14). Neelakantan *et al*. reported 8.98% mandibular second molars with three roots in Indian population which is higher than observation in the present study which is 6.32% of three roots.

In the present study, unilateral occurrence of two roots and three roots (radix entomolaris only) in mandibular second molar were identified in 4 CBCT images (0.58%).

Asians are more likely than other racial groups to have the C-shaped root variant in their mandibular second molars (24). Sri Lanka reported 6.0 % (25), the Sudan reported 10.0 % (26), Saudi Arabia reported 10.6 % (27), Thailand reported 10.9 % 15, and the Burmese population reported 22.4% 14 of C-shaped canal. In the current study, the most prevalent root variation in a mandibular second molar in an Indian population was discovered to be bilateral occurrence of a C-shaped root in 108 CBCT pictures (15.88 %). Seo *et al* reported, the prevalence of the most common root morphology variation, C-shaped canal (31-45%) in a Korean population (28). According to Park *et al*., the incidence rates of bilateral and unilateral C-shaped roots in second molars were 37.0% and 5.6%, respectively, in Korean people (29). Chinese, Japanese, Lebanese, and Hong Kong people have been shown to have a significant occurrence of C-shaped canals (14-52 %) (30-32).

Mandibular first molar with four-rooted morphology has an incidence of 0.04%33, with only three *in vivo* case reports available. No report has been found in literature with incidence of presence of four roots in mandibular second molar (Table 5). In present study the rarest variation found in root morphology was bilaterally presence of four independent roots which was screened in only one CBCT scan image (0.14%). Only one study in Israeli population by Shemesh *et al*. reported 4-rooted mandibular second molars present unilaterally. Extensive search was performed to identify all published case reports of four-rooted mandibular second molars, in the most common electronic databases. Few case reports with unilateral presence of four roots were found (34-36) ( Table 6) reporting similar type of anatomy but none of them showed bilateral presence of four rooted mandibular second molar.

**Table 5:**
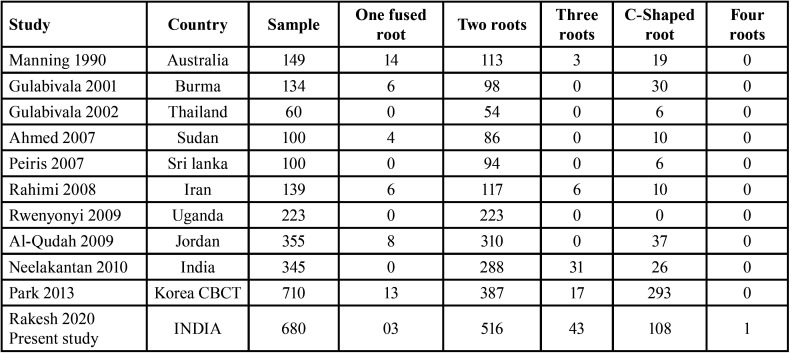
Characterization studies of the mandibular second molar in different populations.

**Table 6:**
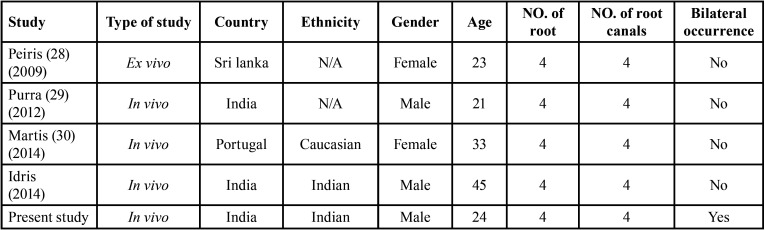
Four-rooted mandibular second molar reports in literature.

While looking for excess or missing root canals, endodontic procedural errors can occur; however, if the clinician is aware of the predicted location, these errors can be reduced. The criteria within which root canal treatment is to be carried out are shown by awareness and understanding of both the normal and aberrant structure of multi-rooted teeth, which improves the success of therapy.

## Conclusions

The mandibular second molar’s most typical root morphology is the bilateral occurrence of two roots and three canals. A less frequent variant is the presence of four bilaterally arranged roots. Knowledge of incidence of rare variations and their unilateral and bilateral occurrence helps the clinician to develop appropriate diagnosis and treatment plans enhancing the success of treatment outcome.

Knowledge of the presence of bilateral or unilateral anatomic variation may help the clinician to develop a diagnosis based on previous treatment of contralateral tooth. CBCT used in the present study for morphological evaluation of root anatomy. Having advantages, it has a has lesser radiation dose, lower scanning time and higher resolution and combination of axial, coronal and sagittal sections, which reduces distortion of images and overlapping of anatomical structures thus providing more accurate anatomy of tooth structure and enhanced visualization of area of interest.
